# Obesity suppresses circulating level and function of endothelial progenitor cells and heart function

**DOI:** 10.1186/1479-5876-10-137

**Published:** 2012-07-02

**Authors:** Tzu-Hsien Tsai, Han-Tan Chai, Cheuk-Kwan Sun, Chia-Hung Yen, Steve Leu, Yung-Lung Chen, Sheng-Ying Chung, Sheung-Fat Ko, Hsueh-Wen Chang, Chiung-Jen Wu, Hon-Kan Yip

**Affiliations:** 1 Division of Cardiology, Department of Internal Medicine; Chang Gung Memorial Hospital- Kaohsiung Medical Center, Chang Gung University College of Medicine, Kaohsiung, Taiwan; 2 Department of Emergency Medicine, E-Da Hospital, I-Shou University, Kaohsiung, Taiwan; 3 Department of Life Science, National Pingtung University of Science and Technology, Pingtung, Taiwan; 4 Center for translational research in biomedical science, Chang Gung Memorial Hospital- Kaohsiung Medical Center, Chang Gung University College of Medicine, Kaohsiung, Taiwan; 5 Departments of Radiology, Chang Gung Memorial Hospital- Kaohsiung Medical Center, Chang Gung University College of Medicine, Kaohsiung, Taiwan; 6 Department of Biological Sciences, National Sun Yat-Sen University Kaohsiung, Kaohsiung, Taiwan; 7 Department of Internal Medicine, Division of Cardiology, Chang Gung Memorial Hospital, Kaohsiung 123, Ta Pei Road, Niao Sung Dist, Kaohsiung City 83301, Taiwan

**Keywords:** Obesity, Aortic endothelium, Endothelial progenitor cells, Angiogenesis, Left ventricular remodeling

## Abstract

**Background and aim:**

This study tested the hypothesis that obesity suppresses circulating number as well as the function of endothelial progenitor cells (EPCs) and left ventricular ejection fraction (LVEF).

**Methods:**

High fat diet (45 Kcal% fat) was given to 8-week-old C57BL/6 J mice (n = 8) for 20 weeks to induce obesity (group 1). Another age-matched group (n = 8) were fed with control diet for 20 weeks as controls (group 2). The animals were sacrificed at the end of 20 weeks after obesity induction.

**Results:**

By the end of study period, the heart weight, body weight, abdominal fat weight, serum levels of total cholesterol and fasting blood sugar were remarkably higher in group 1 than in group 2 (all p<0.01). The circulating level of EPCs (C-kit/CD31, Sca-1/KDR, CXCR4/CD34) was significantly lower in group 1 than in group 2 (p<0.03) at 18 h after critical limb ischemia induction. The angiogenesis and migratory ability of bone marrow-derived EPCs was remarkably impaired in group 1 compared to that in group 2 (all p<0.01). The repair ability of aortic endothelium damage by lipopolysaccharide was notably attenuated in group 1 compared with that in group 2 (p<0.01). Collagen deposition (Sirius red staining) and fibrotic area (Masson's Trichrome staining) in LV myocardium were notably increased in group 1 compared with group 2 (p<0.001). LVEF was notably lower, whereas LV end-diastolic and end-systolic dimensions were remarkably higher in group 1 than in group 2 (all p<0.001).

**Conclusions:**

Obesity diminished circulating EPC level, impaired the recovery of damaged endothelium, suppressed EPC angiogenesis ability and LVEF, and increased LV remodeling.

## Background

Substantial evidence has shown that obesity is commonly associated with a broad range of cardiovascular diseases (CVD), including chronic inflammation, lipid metabolic disorder, accelerated atherosclerosis, increased risk for thrombosis, hypertension, hyperinsulinemia, insulin resistance, and type 2 diabetes mellitus [1-6]. Epidemiological study has further demonstrated that patients with obesity are twice as likely to die of ischemic heart disease compared to those without obesity [7,8]. Indeed, previous studies have identified obesity as an important independent risk factor for cardiovascular events [9-12].

Vascular wall (VW) remodeling from endothelial dysfunction, smooth muscle migration and proliferation, medial-layer thickening, and decreased number of circulating EPCs and VW- resident EPCs in vasa vasorum/adventitia is essential in the development of all stages of atherosclerosis, from initiation, progression, and finally evolvement of complications [13-17]. An association between accumulated CVD risk factors and decreased circulating EPC number [18-21] has been extensively investigated. Additionally, EPCs have been well documented to play an essential role in repairing endothelial function and preventing atherosclerosis [22,23].

Interestingly, while the association between the manifestation of CVD risk factors and decreased circulating number of EPCs [18-21] has been fully investigated, whether obesity itself is an independent factor affecting the number of circulating EPCs is still unclear. Moreover, the impact of obesity on the function of bone marrow-derived (BMD) EPCs has not been studied. Furthermore, of particular significance in translational research is whether obesity suppresses the ability of angiogenesis and vascular repair in response to ischemic stimulation and toxin-induced vascular endothelial damage, respectively.

Therefore, by using a high fat diet-induced murine obesity model, this study aimed at testing the hypotheses that obesity: 1) reduces the number of circulating EPCs, 2) impairs the angiogenic ability of BMDEPCs, 3) hinders the repair of damaged aortic endothelial cells, 4) suppresses left ventricular ejection fraction (LVEF), and 5) aggravates LV remodeling.

## Materials and methods

### Ethics

All animal experimental procedures were approved by the Institute of Animal Care and Use Committee at our institute and performed in accordance with the Guide for the Care and Use of Laboratory Animals (NIH publication No. 85–23, National Academy Press, Washington, DC, USA, revised 1996).

### Animal model of obesity

Eight-week-old male C57J/BL6 mice (n = 18), weighing 22–26 gm, (Charles River Technology, BioLASCO Taiwan Co., Ltd., Taiwan), were fed with high fat diet (45 Kcal% fat; Research Diets, Inc) to obtain the diet-induced obesity model for this study. According to the literature [24] and the instructions from the company (Research Diets, Inc), successful obesity induction in mice was defined as an increase in body weight more than 35% after feeding the animals with high fat diet (45 Kcal% fat) for 13–14 weeks. Therefore, obese mice 20–22 weeks old were utilized for the purpose of this study. By the end of 12 weeks, 18 of 24 mice were observed to fit the criteria of obesity. To determine whether cardiovascular disease develops after prolonged feeding with high fat diet, the 18 obese mice were continuously fed with high fat diet for two additional months (Group 1). For the purpose of the study, another group of aged-matched C57J/BL6 mice (n = 18) purchased from the same company was fed with a control diet for the same period to serve as normal controls (Group 2).

### Animal model of critical limb ischemia for stimulating EPC mobilization into circulation

Mice in groups 1 and 2 were anesthetized by inhalation of 2.0% isoflurane. The mice were placed in a supine position on a warming pad at 37°C with the left hind limbs shaved. Under sterile conditions, the left femoral artery, small arterioles, and circumferential femoral artery were exposed and ligated over their proximal and distal portions before removal. To avoid the presence of collateral circulation, the branches were removed together. Blood was drawn for measuring EPCs using flow cytometry before being sacrificed at 18 h after CLI induction.

### Functional assessment by echocardiography

Transthoracic echocardiography was performed in all animals by a cardiologist blinded to the design of the experiment using a sonographic machine for research purpose (VisualSonics, Vevo, 2100) at the beginning and end of the study with the mice under anesthesia in a supine position. M-mode tracings of left ventricle (LV) were obtained with the heart being imaged in a 2-dimensional mode in long-axis at the level between papillary muscle and mitral valve. Left ventricular internal dimensions [end-systolic diameter (ESD) and end-diastolic diameter (EDD)] were measured according to the American Society of Echocardiography leading-edge method using at least three consecutives cardiac cycles. LVEF was calculated as follows: $\text{LVEF}\;\left(\%\right)=\left[\left({\text{LVEDD}}^{3}-{\text{LVEDS}}^{3}\right)/{\text{LVEDD}}^{3}\right]\times 100$.

### Protocol and assessment of lipopolysaccharide-damaged aortic endothelial cells

This study was designed not only to test the number and function of EPCs, but it also assessed the effect of lipopolysaccharide (LPS) on the severity of aortic endothelial damage and vaso-relaxation impairment in obese and control mice. For this purpose, six mice from each group were randomly chosen to receive LPS treatment.

To elucidate the safety and optimal dosage of LPS for inducing aortic endothelial damage, three dosages of LPS (i.e. 1, 2, and 3 μM intra-peritoneal injection) were given to two mice in each group (i.e. obese and control group). While the highest dosage (3.0 μM) caused mortality, only the moderate dosage was found to cause a significant difference of EPC number in the aortic intimal layer between the two groups of survivors. The moderate dosage of LPS, therefore, was used in the current study.

### Flow cytometric quantification of endothelial progenitor cells

For blood sampling at different time points (i.e. prior to CLI and at 18 h and on day 14 after induction of CLI), cardiac puncture instead of the venous route was adopted for blood sampling using a 30# needle. After treatment with red blood cell-lysing buffer, the cells remained were labeled with appropriate antibodies. Flow cytometric analysis for identification of cell surface markers was performed based on our recent reports [25]. Briefly, the cells were immunostained for 30 minutes with PE-conjugated antibodies against CD31 (BioLegend), KDR (BD Pharmingen), CD34 (BD Pharmingen) and FITC-conjugated antibodies against Sca-1 (BD Pharmingen), c-Kit (BD Pharmingen), CXCR4 (BD Pharmingen). Isotype-identical antibodies (IgG) served as controls. Flow cytometric analyses were performed by utilizing a fluorescence-activated cell sorter (Beckman Coulter FC500 flow cytometer).

Bone marrow mononuclear cell culture, differentiation of endothelial cell phenotype, angiogenesis, and measurement of total tubular length

To evaluate the degree of angiogenesis, bone marrow was drawn from both obese and control animals. The protocol and procedure of cell culture and the determination of angiogenesis were described in our recent report [26]. Briefly, MNCs were isolated by density-gradient centrifugation of Ficoll 400, followed by cultivation in differential endothelial cell culture medium (endothelial cell basal medium-2, Cambrex) with 10% fetal bovine serum (FBS), 50 U/mL penicillin, 50 μg/mL streptomycin and 2 mmol/L L-glutamine (Invitrogen) with vascular endothelial growth factor (VEGF) and basic fibroblast growth factor (10 ng/mL) plated on gelatin-coated tissue culture flasks and incubated at 37°C with 5% CO_2_ for 21 days. Culture medium was changed every 48 hours. By day 21, cells with spindle-shaped and cobblestone-like phenotype typical of endothelial cells were found attached on the plate.

The cells with endothelial cell phenotype were then plated in 96-well plates at 1.0 × 10^4^ cells/well in 150 μL serum-free M199 culture medium mixed with 50 μL cold Matrigel (Chemicon international) for 24 hours using passage 2 EPCs incubated at 37°C in 5% CO_2_. Three random microscopic images (200 ×) were taken from each well for counting cluster, tube, and network formations with the mean values obtained. Both cumulative and mean tube lengths were calculated by Image-Pro Plus software (Media Cybernetics).

### Transwell migratory assay

To investigate the transwell migratory ability of EPCs (n = 6 in each experiment), transwell membranes (5 μm; Costar, Germany) were coated on both sides with fibronectin (2.5 μg/mL; Roche, Mannheim, Germany) overnight at 4 °C. The experimental protocol was also based on that of our recent report [26]. EPCs (1 × 10^5^ cells/well) were resuspended in M199 medium (Gibco, Carlsbad, CA, USA) containing 0.5 FBS (Gibco, Carlsbad, CA, USA) and incubated in the upper chamber at 37°C in 5% CO_2_ and allowed to migrate for 18 hours toward the lower chamber which was filled with M199 containing 20% FBS. Cells remaining on the upper surface of the transwell membranes were mechanically removed and cells that had migrated to the lower surface were fixed with 4% formaldehyde. For cell quantification, nuclei of the migrated cells were stained with DAPI. Cells migrating into the lower chamber were counted over 5 random microscopic fields using a fluorescence microscope (Olympus, Tokyo, Japan) with the software Image-Pro Plus (Media Cybernetics, Bethesda, MD, USA).

### Immunofluorescent (IF) staining

To determine the number of EPCs localized in the endothelial and adventitia (i.e. around the vasa vasorum) layer, IF staining was performed for the examinations of C-kit+, CD34+, Flk-1+ cells (n = 6 for each group) using respective primary antibodies as we previously reported [27]. Irrelevant antibodies were used as controls in the present study.

### Immunohistochemical (IHC) staining

To analyze the extent of collagen synthesis and deposition, three cardiac paraffin sections (6 μm) at 3 mm intervals were stained with picro-Sirius red (1% Sirius red in saturated picric acid solution) for one hour at room temperature using standard methods. The sections were then washed twice with 0.5% acetic acid. The water was physically removed from the slides by vigorous shaking. After dehydration in 100% ethanol thrice, the sections were cleaned with xylene and mounted in a resinous medium. High-power fields (×100) of each section were used to identify Sirius red-positive area on each section. Image-pro plus 6.1 software (Media Cybernetics, Inc., Bethesda, MD, USA) was used to calculate the total area of Sirius red-positive staining. The mean area of collagen deposition was obtained by summation of Sirius red-positive areas on each section divided by the total numbers of sections.

### Histological study of fibrosis area in LV

Masson's Trichrome staining was used for studying fibrosis of LV myocardium. The integrated area (μm^2^) of fibrosis in LV myocardium in the tissue sections was calculated using Image Tool 3 (IT3) image analysis software (University of Texas, Health Science Center, San Antonio, UTHSCSA; Image Tool for Windows, Version 3.0, USA) as described previously [27]. Three selected sections were quantified for each animal. Three randomly selected high-power fields (HPFs) (400 ×) were analyzed in each section. After determining the number of pixels in each fibrosis area per HPF, the numbers of pixels obtained from the three HPFs were summated. The procedure was repeated in two other sections for each animal. The mean pixel number per HPF for each animal was then determined by summating all pixel numbers and dividing by 9. The mean area of fibrosis per HPF was obtained using a conversion factor of 19.24 (1 μm^2^ represented 19.24 pixels).

### Measurement of aortic contractility and nitric oxide release on LPS overload

At the end of the study, six mice in each group were treated by LPS. Forty-eight hours after LPS treatment, the mice were sacrificed and the aorta in each mouse was isolated. Aortic segments in each mouse were collected for identification of EPCs localized in aortic endothelial layer using IHC staining. Other aortic segments were cleaned and cut into slices of 2 mm in length for evaluating the contractile and relaxant response as previously reported [28] with some modifications. Briefly, the aortic rings were carefully mounted on an isometric force transducer (XDFT05, Singa, Taiwan) with a tension of 1.8 g and placed in an organ chamber filled with Krebs solution (NaCl, 99.01 mM; KCl, 4.69 mM; CaCl_2_, 1.87 mM; MgSO_4_, 1.20 mM; K_2_HPO_4_, 1.03 mM; glucose, 11.1 mM) maintained at pH 7.4 and bubbled with 95% O_2_ - 5% CO_2_. After an equilibration of 40 minutes, 1 μM of phenylephrine (PE) was added to the organ chamber for the assessment of contractile activity, and then 30 μM of acetylcholine (ACh) was added to assess the endothelial integrity. After washing and a re-equilibration for 30 minutes, a cumulative PE dose (from 1 nM to 1 μM) was added to the organ chamber to obtain a concentration-dependent contractile curve, and then sodium nitroprusside (30 μM) was added to the organ chamber to obtain a relaxant response. After washing and a re-equilibration for 20 minutes, 30 μM of ACh was added into the organ chamber, followed by 1 μM of PE to evaluate the endothelium-dependent vasorelaxant response. Then PE (1 μM)-induced vasocontractile response was assessed again in the presence of L-NAME (100 μM) pre-treatment for 30 minutes. All data were acquired and analyzed using the XctionView system (XctionView, Singa, Taiwan).

### Protocol for assessment of aortic basal nitric oxide (NO) release on LPS overload

Vascular basal nitric oxide release was calculated as the percentage of difference between PE-induced vasocontractile response in the absence and presence of L-NAME according to our previous study [28].

### Statistical analysis

Quantitative data are expressed as means ± SD. Statistical analysis was adequately performed by ANOVA followed by Tukey’s multiple comparison procedure. Statistical analysis was performed using SAS statistical software for Windows version 8.2 (SAS institute, Cary, NC). A probability value <0.05 was considered statistically significant.

## Results

### Baseline characteristics, laboratory findings, and transthoracic echocardiography results

The initial body weight (BW) did not differ between the obese (group 1) and control (group 2) animals. However, the final BW was significantly higher in group 1 than in group 2. Neither the heart weight nor the ratio of heart weight to tibial bone length was different between the two groups. Additionally, the initial blood sugar did not differ between group 1 and group 2. However, the abdominal fat weight, serum levels of total cholesterol and final blood sugar were notably higher in group 1 than in group 2 (Table 1).The initial LVEF, fractional shortening (FS), LVEDd, LVESd and the thickness of interventricular septum and posterior wall did not differ between groups 1 and 2. Moreover, at the end of the study, the thickness of interventricular septum and posterior wall were similar between groups 1 and 2. However, at the end of study, LVEF and fractional shortening were significantly lower, whereas LVEDd and LVESd were remarkably higher in group 1 than in group 2 (Table 1).

**Table 1:** The baseline characteristics, laboratory findings and echocardiography results

**Variables**	**Obesity (n = 8)**	**Control (n = 8)**	P value*
Initial body weight (g)	25.7 ± 0.66	26.0 ± 0.72	0.448
Final body weight (g)	43.9 ± 1.55	34.4 ± 1.48	<0.0001
Heart weight (g)	0.19 ± 0.02	0.14 ± 0.01	<0.0001
Ratio of heart weight to tibial bone length	0.095 ± 0.002	0.070 ± 0.001	<0.001
Abdominal fat weight (g)	4.09 ± 0.65	1.66 ± 0.41	<0.0001
Total cholesterol (mg/dl)	232.8 ± 7.53	136.3 ± 11.8	<0.01
Initial fasting blood glucose level (mg/dl)†	103 ± 8.63	98.3 ± 9.0	0.657
Final fasting blood glucose level (mg/dl)†	307.5 ± 74.7	142.5 ± 18.3	<0.0001
Circulating level of endothelial progenitor cells			
C-kit/CD31 (%)			
Baseline (0 h)‡	1.15 ± 0.21	1.06 ± 0.40	0.636
18 hour§	2.60 ± 0.49	3.29 ± 0.50	0.025
Sca-1/KDR (%)			
Baseline‡	0.85 ± 0.27	0.66 ± 0.21	0.169
18 hour§	1.05 ± 0.24	1.60 ± 0.22	0.042
CXCR4/CD34 (%)			
Baseline (0 h)‡	0.38 ± 0.17	0.43 ± 0.27	0.745
18 hour§	0.68 ± 0.31	1.13 ± 0.36	0.033
Echocardiograph (baseline)			
Inter-ventricular septum (mm)	0.82 ± 0.08	0.83 ± 0.09	0.360
Posterior wall (mm)	0.77 ± 0.11	0.76 ± 0.09	0.601
Left ventricular end-diastolic dimension (mm)	4.10 ± 0.10	3.93 ± 0.26	0.438
Left ventricular end-systolic dimension	2.71 ± 0.21	2.63 ± 0.23	0.588
Fractional shortening (%)	32.7 ± 1.98	34.4 ± 2.01	0.212
Left ventricular ejection fraction (%)	62.5 ± 2.43	64.0 ± 2.49	0.327
Echocardiograph (at end of study period)			
Inter-ventricular septum (mm)	0.87 ± 0.06	0.86 ± 0.08	0.949
Posterior wall (mm)	0.79 ± 0.08	0.77 ± 0.03	0.686
Left ventricular end-diastolic dimension (mm)	4.46 ± 0.17^b^	4.18 ± 0.16^a^	0.031
Left ventricular end-systolic dimension	3.46 ± 0.48^b^	2.78 ± 0.13^a^	<0.001
Fractional shortening (%)	22.8 ± 1.15^b^	33.4 ± 0.85^a^	<0.001
Left ventricular ejection fraction (%)	46.0 ± 1.95^b^	62.7 ± 1.43^a^	<0.001

Group 1 = obesity.

Group 2 = normal control.

* by *t* test.

† value was observed after 12 fasting in the mice.

‡ blood sample was collected prior to critical limb ischemia procedure.

§ blood sample was collected at 18 h after critical limb ischemia procedure.

The initial LVEF, fractional shortening (FS), LVEDd, LVESd and the thickness of interventricular septum and posterior wall did not differ between groups 1 and 2. Moreover, at the end of the study, the thickness of interventricular septum and posterior wall were similar between groups 1 and 2. However, at the end of study, LVEF and fractional shortening were significantly lower, whereas LVEDd and LVESd were remarkably higher in group 1 than in group 2.

### Circulating level of EPC at baseline and 18 h after critical limb ischemia (CLI) procedure

Interestingly, at day 0 prior to the procedure, the circulating levels of EPCs (C-kit/CD31, Sca-1/KDR, CXCR4/CD34) were similar between group 1 and group 2. However, at 18 h after the CLI procedure, these biomarkers were notably lower in group 1 than in group 2 (Table 1). These findings implicate that EPC mobilization from bone marrow to circulation in response to ischemic stimuli was markedly impaired in obesity.

### IHC and IF staining of aorta

**Figure 1:**
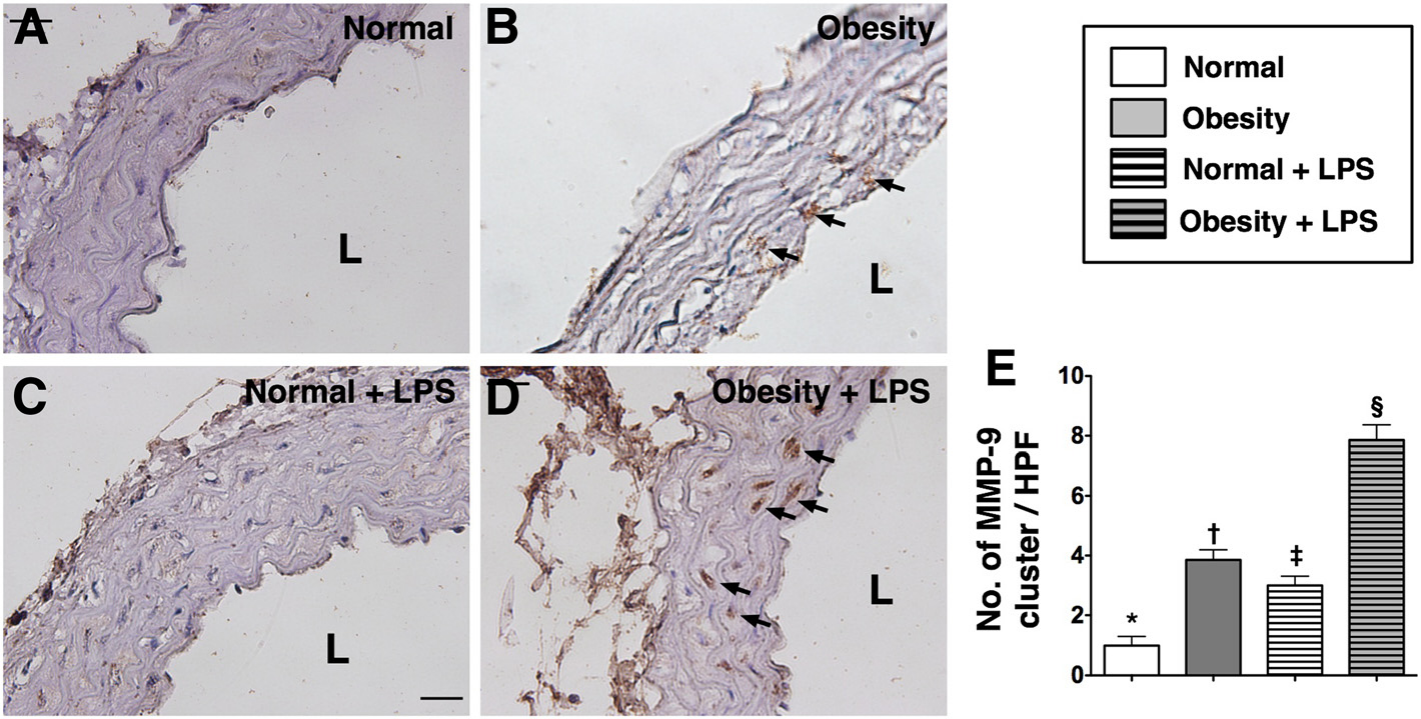
**Immunohistochemical (IHC) staining (400x) for the cluster formation (≥ 2.0 μm) of matrix metalloproteinase (MMP)-9 in medial layer of aorta (n = 6). E) **Showing the cluster formation of MMP-9 (black arrows) was significantly higher in obesity (**B**) than in normal control (**A**) prior to lipopolysaccharide (LPS) treatment, more significantly higher in obesity (**D**) than in normal control (**C**) after LPS treatment. Statistical analysis by one-way ANOVA. * vs. † vs. ‡ vs. §, p < 0.0001. Symbols (*, †, ‡, §) indicate significant difference (at 0.05 level) followed by Bonferroni’s multiple-comparisons post hoc test. L = lumen side. Scale bars in upper or lower corner represent 20 μm. HPF = high-power field.

Prior to LPS treatment, IHC staining showed that the number of patched distribution (diameter ≥ 2.0 μm) of MMP-9 in medial layer of aorta, an index of inflammation, was remarkably higher in group 1 than in group 2. In addition, at 18 hours after LPS treatment, the number of patched distribution of these biomarkers in the medial layer of the aorta was remarkably higher in group 1 than in group 2. On the other hand, the number of patched distribution of this biomarker did not differ among group 2 animals with and without LPS treatment (Figure 1).

**Figure 2:**
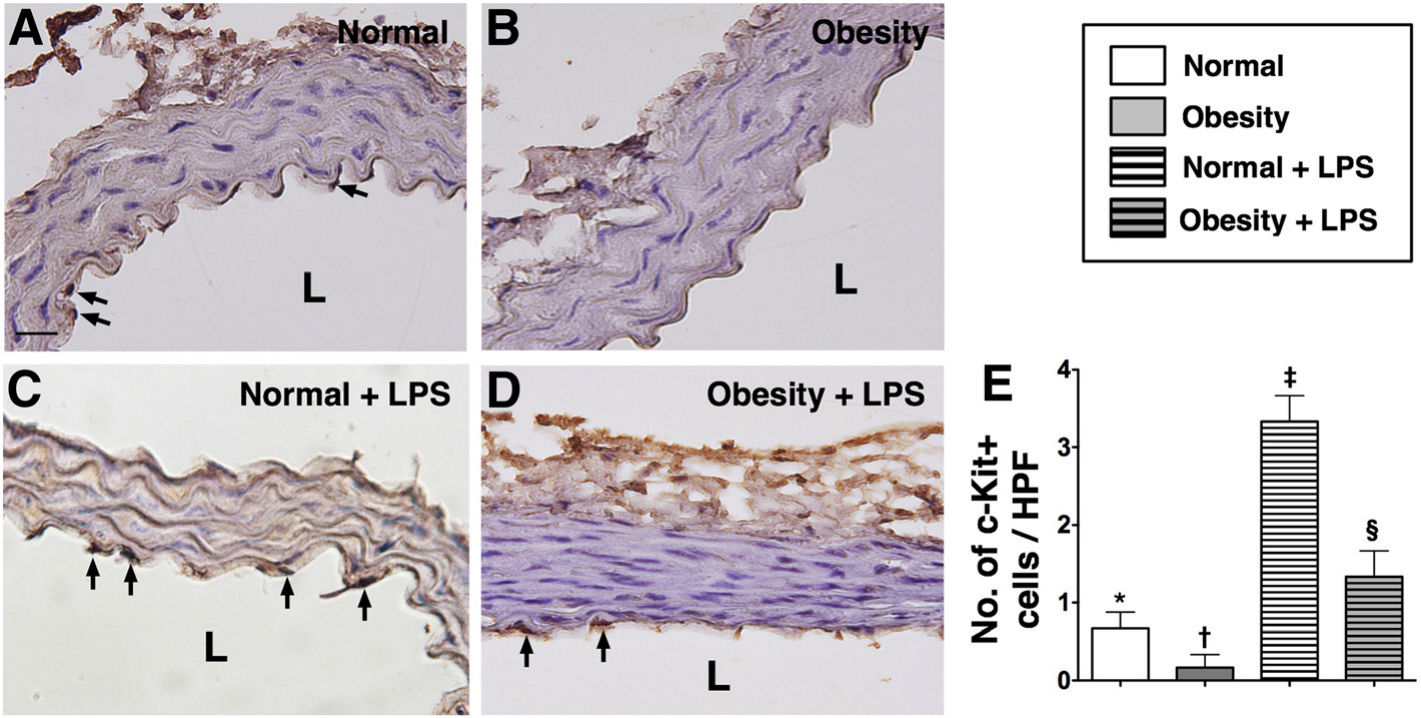
**IHC staining (400x) for number of C-kit + stained cells in endothelial layer of aorta (n = 6). E**) Demonstrating the number of C-kit + stained cells (black arrows) was significantly higher in normal control (**A**) than in obesity (**B**) prior to LPS treatment, more significantly higher in normal control (**C**) than in obesity (**D**) after LPS treatment. Statistical analysis by one-way ANOVA. * vs. † vs. ‡ vs. §, p < 0.0001. Symbols (*, †, ‡, §) indicate significant difference (at 0.05 level) followed by Bonferroni’s multiple-comparisons post hoc test. L = lumen side. Scale bars in left lower corner represent 20 μm. HPF = high-power field.

The numbers of C-kit + and Sca-1+ cells located in the intimal layer of aorta were significantly higher group 2 than in group 1 prior to LPS treatment, whereas the number of Flk-1+ cells in the intimal layer of aorta was similar between the two groups. However, at 18 h after LPS treatment, the numbers of cells positive for these biomarkers in the intimal layer of aorta were substantially higher in group 2 than in group 1 (Figure 2, 3 &4). These findings implicate that obesity impairs the ability of recovery after endotoxin-induced damage of endothelial cells.

**Figure 3:**
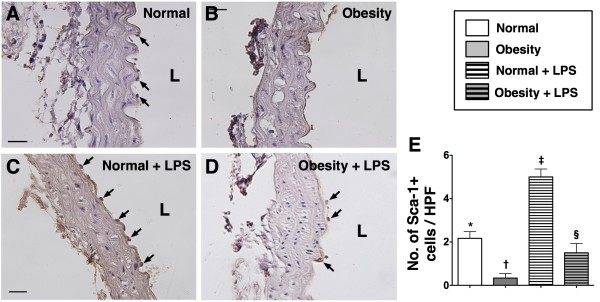
**IHC staining (400x) for number of Sca-1+ stained cells in endothelial layer of aorta (n = 6). E**) Demonstrating the number of Sca-1+ stained cells (black arrows) was significantly higher in normal control (**A**) than in obesity (**B**) prior to LPS treatment, more remarkably higher in normal control (**C**) than in obesity (**D**) after LPS treatment. Statistical analysis by one-way ANOVA. * vs. † vs. ‡ vs. §, p < 0.0001. Symbols (*, †, ‡, §) indicate significant difference (at 0.05 level) followed by Bonferroni’s multiple-comparisons post hoc test. L = lumen side. Scale bars in lower or upper corner represent 20 μm. HPF = high-power field.

**Figure 4:**
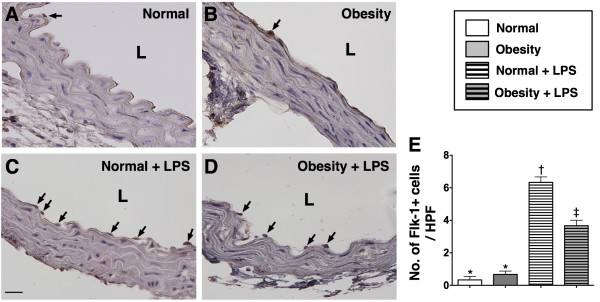
**IHC staining (400x) for number of Flk-1+ stained cells in endothelial layer of aorta (n = 6). E**) Demonstrating the number of Flk-1+ stained cells (black arrows) was similar between normal control (**A**) and obesity (**B**) prior to LPS treatment. However, the number of this biomarker in endothelial layer of aorta was significantly higher in normal control (**C**) than in obesity (**D**) after LPS treatment. Statistical analysis by one-way ANOVA. * vs. † vs. ‡, p < 0.0001. Symbols (*, †, ‡) indicate significant difference (at 0.05 level) followed by Bonferroni’s multiple-comparisons post hoc test. L = lumen side. Scale bars in left lower corner represent 20 μm. HPF = high-power field.

### Satining of left ventricular myocardium

**Figure 5:**
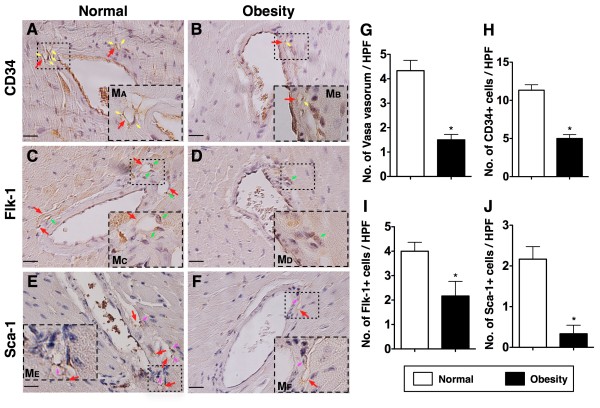
**IHC staining (400x) for number of the vasa vasorum and numbers of CD34+, Flk-1+, Sca-1+ cells in left ventricular myocardium (n = 8). G**) The number of vasa vasorum (red arrows) was remarkably higher in normal control than in obesity. * vs. normal, p < 0.0001. **H**) The number of CD34+ stain cells (yellow arrows) was significantly higher in normal control (**A**) than in obesity (**B**). * vs. normal, p < 0.001. **I**) The number of Flk-1+ cells (green arrows) was notably higher in normal control (**C**) than in obesity (**D**). * vs. normal, p < 0.001. **J**) The number of Sca-1+ cells (pink arrows) was notably higher in normal control (**E**) than in obesity (**F**). * vs. normal, p < 0.001. M_A,_ M_B_, M_C_, M_D_, M_A_, and M_AF_ indicated dotted line area (i.e., the vasa vasorum) was magnified for 1000x. Scale bars in left lower corner represent 20 μm.

Hematoxylin and eosin (H & E) staining revealed no evidence of atherosclerotic obstructive coronary artery (CAD) in both obese and normal control mice (Figure 5-A to 5-F). However, the number of vasa vasorum (Figure 5-G) and EPCs [CD34+ (Figure 5-H), Flk-1+ (Figure 5-I), Sca-1+ (Figur 5-J)] around vasa vasorum/adventia of large epicardial vessels were remarkably reduced in group 1 as compared with group 2.

### Sirius red staining for collagen deposition and Masson's trichrome staining for fibrosis in left ventricular myocardium

**Figure 6:**
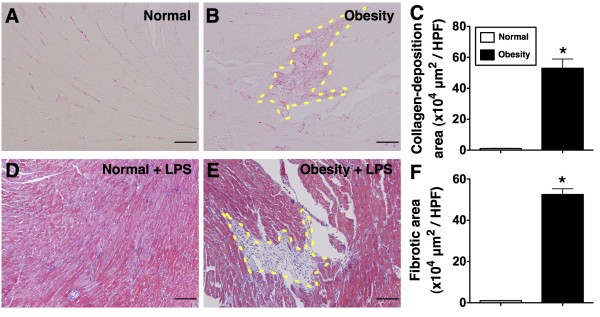
**Sirius red stain and Masson's Trichrome Stain of left ventricular (LV) myocardium (n = 8). C**) The results of Sirius red staining (100x) demonstrated that the collagen-fiber deposition area (yellow-dot lines) in LV myocardium was significantly higher in obesity (**B**) than in normal control (**A**). **F**) The results of Masson's trichrome staining (100x) showed significantly higher fibrotic area in obesity (**E**) than in normal control (**D**). * vs. normal, p < 0.0001. Scale bars in left lower corner represent 100 μm. HPF = high-power field.

Sirius red staining (Figure 6-A & 6-B) demonstrated that collagen fiber deposition in LV myocardium was remarkably higher in group 1 than in group 2 (Figure 6-C). In addition, Masson's Trichrome staining (Figure 6-D & 6-E) revealed that the distribution of fibrosis was markedly increased in group 1 animals compared to that in group 2 (Figure 6-F).

### Aortic vasorelaxation and NO release

**Figure 7:**
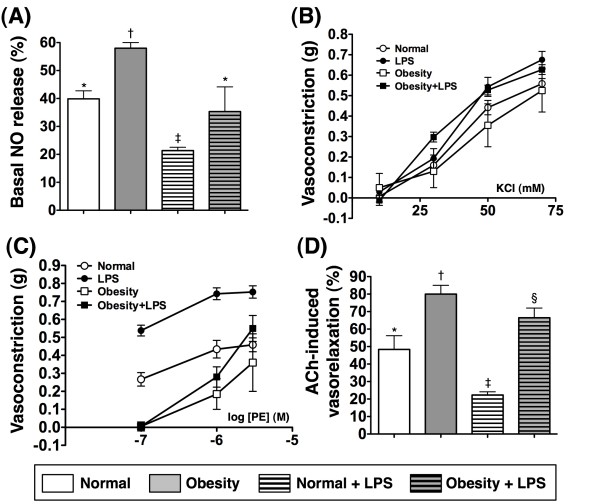
**Aortic vasorelaxation and nitric oxide (NO) release (n = 6). A**) NO release from endothelial cells of aorta was significantly higher in obesity than in normal control prior to LPS treatment. NO-release was remarkably impaired in obesity and more remarkably impaired in normal control after LPS treatment. * vs. † vs. ‡ vs. §, p < 0.0001. **B**) the KCl-induced vasoconstriction did not differ in obesity as compared with normal control regardless with and without LPS treatment. **C**) Phenylephrine (PE)-induced vasoconstriction was notably reduced in obesity as compared with normal control prior to LPS treatment (p < 0.01). Additionally, after the LPS treatment, the responsibility of PE-induced vasoconstriction was more prominent in normal control than in obesity (p < 0.001). **D**) Ach-induced vasorelaxation was significantly lower in normal control than in obesity prior to LPS treatment and more significantly lower in normal control than in obesity after LPS treatment (all p values < 0.001). * vs. † vs. ‡ vs. §, p < 0.0001. Symbols (*, †, ‡, §) indicate significant difference (at 0.05 level) followed by Bonferroni’s multiple-comparisons post hoc test.

Baseline NO release from endothelial cells of aorta was significantly enhanced in group 1 compared to that in group 2. Moreover, after LPS-induced endothelial damage, the NO-releasing ability was remarkably impaired in both groups, particularly in group 2 (Figure 7-A). These findings suggest that overactive NO production may occur in obese mice to maintain vasorelaxation at both baseline and in situation of LPS-induced endothelial damage.

Baseline KCl-induced vasoconstriction did not differ in obesity as compared with normal control regardless with and without LPS treatment (Figure 7-B). Baseline PE-induced vasoconstriction was notably reduced in group 1 compared to that in group 2. Besides, after LPS treatment, the responsibility of PE-induced vasoconstriction was weaker in group 1 than in group 2. By contrast, ACh-induced vasorelaxation exhibited an opposite manner compared to that of PE-induced vasoconstriction in baseline condition and after LPS treatment in both groups of animals (Figure 7-C & 7-D). The results indicate that the VW of obese mice was in a hyper-reactive state toward both vasodilatation and vasoconstrictive stimuli.

### In vitro studies for measurement of total tubular length, transwell migratory assay, and proliferation assessment

**Figure 8:**
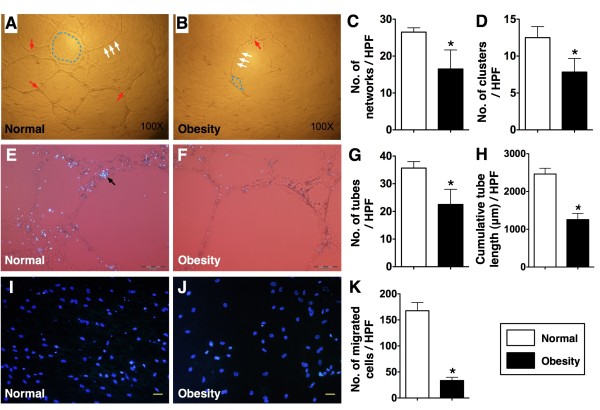
**Angiogenesis and migratory assay (n = 6). **Illustration (100x) of angiogenesis in normal control (**A**) and obesity (**B**). The network (blue-dot lone) (**C**), cluster (red arrow) (**D**) and tubular (white arrows) (**G**) formation and tubular length (**H**), indexes of angiogenesis, were significantly increased in normal control than in obesity. **E & F**) Human umbilical vein endothelial cells (HUVECs) were utilized for formation of angiogenesis platform (100×). The same number of EPCs (1.0 × 10^3^) (DAPI positively stained cells) from normal control and obesity mice were co-cultured with HUVECs (2.0 × 10^3^) in Matrigel for 24 h. The results showed notably higher number of EPCs and cluster formation (black arrow) within the HUVEC-formed angiogenic platform in normal than in obesity. Scale bar in right lower corner represent 100 μm. **I & J**) Illustration of migratory assay (400×). The blue color represented the nuclei were stained with DAPI. Scale bar in right lower corner represent 20 μm. **K**) Showing the numbers of migratory EPCs in Transwell, an index of EPC kinesis, were significantly higher in normal control than in obesity. For (**D**), * vs. normal, p < 0.001; For (**C**), (**G**), (**H**) and **(K**), * vs. normal, p < 0.0001.

To compare the angiogenesis ability and function of EPC between obesity and normal mice, BMDMNC culture for EPC was performed. Interestingly, the results showed that cluster formation (p < 0.001), tubular formation (p < 0.0001), and network formation (p < 0.0001) were remarkably lower in obese mice than in control mice (Figure 8-A to 8-F). In addition, the accumulative tubular length (P < 0.0001) was also notably reduced in obese mice. Furthermore, the number of transwell migratory EPCs (Figure 8-I & 8-J ) was substantially lower in obese mice compared to that in control mice (p<0.0001).

## Discussion

This study, which investigated the impact of obesity on cardiovascular pathogenesis in a murine obesity model, yielded several striking implications. First, repair ability of EPC in response to LPS-induced damage of aortic endothelium was remarkably impaired in obese animals. Second, the circulating level of EPCs in the setting of CLI was significantly reduced in obese mice compared to that in normal mice. Third, LV remodeling was markedly increased, whereas LV function was notably suppressed in obese mice compared with the normal control animals. Fourth, hyper-reactive vasorelaxation and over compensatory NO production from aorta was observed in the obese animals. Finally, the number of EPCs and vasa vasorum in adventitia layer of epicardial arteries was significantly reduced in the setting of obesity.

In the present study, not only does the entire body weight increase, but also heart weight, ratio of heart weight to tibial length, abdominal fat weight, and total cholesterol level were substantially higher in obese animals than those in control animals. These findings suggest that the high-fat diet induced a successful obesity model in mice. One important finding is that feeding with high-fat diet also induced hyperglycemia.

Clinical observational studies have previously shown that the circulating number of EPCs was significantly decreased in manifestation of CVD risk factors and the presence of coronary artery disease (CAD) [18-21]. Interestingly, in the present study, histological analysis of the LV sections with α-SMA staining showed no evidence of atherosclerotic obstructive CAD in obese mice. Additionally, the baseline circulating levels of EPC were similar between obese and normal control mice. Our results, therefore, in addition to supporting the findings of previous studies [18-21], also provide useful information that the circulating number of EPCs may be normal in the setting of obesity without manifestation of atherosclerotic obstructive CAD.

The link between obesity and endothelial and vascular dysfunction has been extensively discussed recently [29,30]. An essential finding in the current study is that, as compared with the normal controls, an increase in the circulating number of EPCs in response to ischemic stress was remarkably suppressed in obese mice. This finding indicates that the EPC mobilization ability from bone marrow to circulation in response to ischemic stimulation is curtailed in obesity. Moreover, aortic endothelial repair ability after LPS-induced endothelial damage was found to be notably reduced in obese animals. Furthermore, Matrigel assay demonstrated that the angiogenesis ability, an index of endothelial function, was substantially suppressed in the setting of obesity. Moreover, migratory assay identified that the EPC migratory ability was significantly hindered in obese animals compared to that in their normal counterparts. These findings highlight the fact that EPC/endothelial function (i.e., angiogenesis, EPC kinetics) rather than EPC number is reduced before the manifestation of obstructive CAD. In this way, our findings reinforce those of previous clinical observational studies [29,30].

Although the role of obesity in impairing LV function and increasing LV remodeling have been keenly investigated in previously clinical observational studies, the study of heart function in animal models of obesity has seldom been reported [31-33]. The most important finding in this experimental study is that LVEF and FS were significantly lower in obese mice than those in normal controls. Of importance is that LV remodeling (i.e., increased LVEDd and LVESd) was remarkably increased in obese animals compared to that in their normal counterparts. Accordingly, the results of the present study support the findings from previous clinical observational studies [31-33]. Another principal finding in the current study using Masson's Trichrome staining showed that the fibrotic area was substantially increased in obese mice. Consistently, Sirius red staining revealed significantly heavier deposition of collagen fiber in LV myocardium of obese mice than that of normal controls. Furthermore, the numbers of vasa vasorum and EPCs over adventitia of epicardial vessels were significantly reduced in the former than in the latter animals. These findings may, at least in part, explain the notable impairment in LV function significant increase in LV remodeling in the obese mice even in the absence of atherosclerotic obstructive CAD (i.e. α-SMA results of patent epicardial vessels). Surprisingly, the underlying mechanisms involved in cardiac remodeling in obese patients without overt atherosclerosis-related CAD have not been investigated in previous clinical observational studies [31-33]. Our results, therefore, further extend the findings of those studies [31-33].

On the other hand, previous studies have emphasized that obesity results in an imbalance between endothelium-derived vasoactive factors favoring vasoconstriction and inflammatory activation. Abnormal regulation of these factors due to endothelial cell dysfunction is both the consequence and cause of vascular disease processes [34]. Thus, obesity can be considered to cause accelerated "premature" vascular aging [34]. Interestingly, the results of the present study demonstrated notably increased baseline NO release from the aorta in obese mice than that in the normal controls (Figure 7-A). Additionally, NO release after LPS-induced aortic endothelial damage was still significantly higher in the former than in the latter (Figure 7-A). Based on this finding, we propose that this phenomenon may be due to 1) a compensatory response to obesity-induced endothelial dysfunction at the early stage in a scenario comparable to hyper-insulinemic response in the setting of early hyperglycemic/diabetes mellitus situation; 2) it may also be due to a relatively higher intrinsic resistance of murine aortic endothelial cells against obesity-induced damage compared to that in the human beings. This second hypothesis could be further supported by the finding (Figure 7-B) that KCl-induced vasoconstriction (i.e. the integrity of calcium channel in smooth muscle cells) did not differ between obese and normal control mice. Our findings could further explain the notably lower PE-induced vasoconstriction (Figure 7-C), but apparently higher the ACh-induced vasorelaxation (Figure 7-D) in obese mice than those in normal controls. In this way, our findings are not completely identical to the those previously reported [34,35].

### Study limitations

This study has limitations. First, the exact underlying mechanisms involved in the impairment of heart function were not fully investigated in the current study. Second, the effects of therapeutic intervention and statin treatment on body weight, hypercholesterolemia, heart function, and circulating level of EPCs were not examined in the present study.

## Conclusions

This study demonstrated that the circulating number and function of EPCs as well as heart function were markedly reduced, whereas LV remodeling was significantly enhanced in obesity even without overt atherosclerotic obstructive CAD.

## Competing interests

The authors declare that they have no competing interests.

## Authors’ contributions

All authors have read and approved the final manuscript. YKH, TTH, CHT, WCJ and SL designed the experiment, drafted and performed animal experiments. SCK, SS, CYL, CSY, CHW and KSF were responsible for the laboratory assay and troubleshooting. YKH, WCJ and HKY participated in refinement of experiment protocol and coordination and helped in drafting the manuscript. All authors report no disclosures and have any commercial associations or interests, including consultancies, stock ownership or other competing equity interest.
